# Endothelial Dysfunction and Specific Inflammation in Obesity Hypoventilation Syndrome

**DOI:** 10.1371/journal.pone.0006733

**Published:** 2009-08-24

**Authors:** Jean-Christian Borel, Pascale Roux-Lombard, Renaud Tamisier, Claire Arnaud, Denis Monneret, Nathalie Arnol, Jean-Philippe Baguet, Patrick Levy, Jean-Louis Pepin

**Affiliations:** 1 INSERM ERI17, Laboratoire HP2, Université Joseph Fourier, Faculté de Médecine, Grenoble, France; 2 CHU, Hôpital A. Michallon, Pôle Rééducation et Physiologie, Grenoble, France; 3 Service d'Immunologie et d'Allergologie, Hôpitaux Universitaires et Université de Genève, Genève, Suisse; 4 CHU, Hôpital A. Michallon, Service de cardiologie, Grenoble, France; Lerner Research Institute, Cleveland Clinic, United States of America

## Abstract

**Background:**

Obesity hypoventilation syndrome (OHS) is associated with increased cardiovascular morbidity. What moderate chronic hypoventilation adds to obesity on systemic inflammation and endothelial dysfunction remains unknown.

**Question:**

To compare inflammatory status and endothelial function in OHS *versus* eucapnic obese patients.

**Methodology:**

14 OHS and 39 eucapnic obese patients matched for BMI and age were compared. Diurnal blood gazes, overnight polysomnography and endothelial function, measured by reactive hyperemia peripheral arterial tonometry (RH-PAT), were assessed. Inflammatory (Leptin, RANTES, MCP-1, IL-6, IL-8, TNFα, Resistin) and anti-inflammatory (adiponectin, IL-1Ra) cytokines were measured by multiplex beads immunoassays.

**Principal Findings:**

OHS exhibited a higher PaCO_2_, a lower forced vital capacity (FVC) and tended to have a lower PaO_2_ than eucapnic obese patients. _HS_-CRP, RANTES levels and glycated haemoglobin (HbA1c) were significantly increased in OHS (respectively 11.1±10.9 vs. 5.7±5.5 mg.l^−1^ for _HS_-CRP, 55.9±55.3 *vs* 23.3±15.8 ng/ml for RANTES and 7.3±4.3 vs 6.1±1.7 for HbA1c). Serum adiponectin was reduced in OHS (7606±2977 *vs* 13660±7854 ng/ml). Endothelial function was significantly more impaired in OHS (RH-PAT index: 0.22±0.06 vs 0.51±0.11).

**Conclusions:**

Compared to eucapnic obesity, OHS is associated with a specific increase in the pro-atherosclerotic RANTES chemokine, a decrease in the anti-inflammatory adipokine adiponectin and impaired endothelial function. These three conditions are known to be strongly associated with an increased cardiovascular risk.

**Trial Registration:**

ClinicalTrials.gov NCT00603096

## Introduction

The obesity-hypoventilation syndrome (OHS) is defined by obesity (BMI≥30 kg/m^2^), and chronic alveolar hypoventilation resulting in daytime hypercapnia (PaCO_2_>45 mmHg), after exclusion of all other causes of alveolar hypoventilation (severe obstructive or restrictive diseases, chest wall disorders, neuromuscular diseases)[1]–[3]. Patients suffering from OHS are considered to be more severely affected compared to eucapnic obese patients. The use of health-care resources is increased compared to usual obese patients [4] and OHS is carrying a momentous cardiovascular morbidity [2], [5]. Compared with obese control subjects, patients with OHS are statistically much more likely to have been diagnosed with congestive heart failure (OR 9; 95% CI, 2.3–35), angina pectoris (OR, 9; 95% CI, 1.4–57.1) and cor pulmonale (OR, 9; 95% CI, 1.4–57.1) [4]. In obese, prospectively followed during 18 months after hospital discharge, OHS patients had a higher rate of death compared to simple obesity (23% versus 9%) [6].

Obesity is a disease state characterized by chronic systemic low grade inflammation and associated inflammatory changes in the adipose tissue [7]–[10]. OHS adds up on obesity several extra stimuli that might increase the burden of chronic inflammation and as a consequence its proatherogenic effects. Work of breathing is higher in OHS compared to eucapnic obesity [11]. It has been demonstrated that adding a load to the respiratory system is resulting in proinflammatory cytokines release [12], [13]. Up to 85% percent of OHS patients are exhibiting sleep apnea, a disease condition, linked with cardiovascular diseases [14]. Furthermore, OHS is characterized by mild hypoxaemia during daytime, associated with extreme oxygen desaturations during REM sleep and concomitant repeated acute increases in PaCO_2_. In animal models and humans, hypoxia of the adipose tissue has been shown to be associated with local fat inflammation[15], [16]. Thus, an aggravation in adipose tissues of inflammatory state is conceivable in OHS. Finally, the pivotal mechanism underlying daytime hypercapnia in OHS is the reduction in ventilatory drive owing to central leptin resistance [17], [18]. Central leptin resistance results in high plasmatic levels of leptin and preservation of peripheral actions of leptin such as increased sympathetic outflow and cytokine production [19].

Thus, it seemed reasonable to hypothesize that OHS compared to eucapnic obesity could be associated with increased systemic inflammation and with an increased production of proinflammatory adipocytokines. Furthermore Apovian et al. have recently demonstrated that local adipose tissue inflammation is linked with endothelial dysfunction [7]. We postulated that OHS patients would exhibit a specific inflammatory response and a more severe endothelial dysfunction than eucapnic obese, matched for BMI and age.

## Materials and Methods

### Patients

Obesity hypoventilation syndrome (OHS) was defined by a body mass index (BMI) above 30 kg/m^2^ and a PaCO_2_>6 kPa on daytime blood gazes, without any significant airway obstruction (FEV1/FVC<70%), history of heart failure or progressive neuromuscular disease. OHS patients were compared to control subjects (eucapnic obesity group), in a case control study design. Both groups of subjects came from an obese database of subjects recruited by advertisement in newspapers or addressed to the sleep laboratory for suspicion of obstructive sleep apnea syndrome (OSAS). Among 104 obese in the database at the time of the analysis, each OHS patient was matched with up to 6 eucapnic obese. Patients were matched for two classes of age (≤60 or>60 years old), and three classes of body mass index (30≤BMI<35 kg.m^−2^), (35≤IMC<40 kg.m^−2^), (BMI>40 kg.m^−2^). All the patients underwent a baseline screening visit including sleep studies, respiratory assessment and cardiovascular function before been potentially involved in a randomized interventional controlled study. After this baseline visit, only those subjects having obesity hypoventilation syndrome were randomized in a one month comparison between non invasive ventilation and standard care. This randomized controlled study is ongoing [clinical trial registration number: NCT00603096]. The current study is reporting the results of a case control comparison of two clusters of obese exhibiting or not OHS and matched for age and BMI.

The study was approved by the university hospital ethics committee. All patients signed a written informed consent.

### Study design

Patients underwent an overnight polysomnography. After waking up, in fasting state, a peripheral blood sample was drawn and endothelial dysfunction was assessed by reactive hyperhemia with finger plethysmographic methodology (RH-PAT). After breakfast, Epworth sleepiness scale, pulmonary function tests, arterial blood gases analysis, and ventilatory response to CO_2_ were performed.

### Study procedures

#### Polysomnography (PSG)

An overnight PSG was performed during spontaneous breathing in order to characterize abnormal respiratory events during sleep according to standard criteria [20], [21] as previously described [22], [23].

#### Biomarkers

After peripheral blood sampling, plasma glucose and serum triglycerides levels were measured on automat (Modular 700, Roche, Meylan, France). Serum insulin was measured using a radio-immunometric sandwich assay (CIS bio international, Gif-Sur-Yvette, France). Serum HS-CRP level was measured using automated immunonephelometry (Behring Nephelometer II Analyzer, Dade Behring, Germany).

Leptin, CCL5/RANTES (Regulated upon activation normal T-cell express an secreted), CCL2/MCP1 (Monocyte chemo-attractant protein 1), IL-6, IL-8, TNFα, Resistin, Adiponectin and IL-1Ra were measured by commercially available multiplex beads immunoassays (Fluorokine MAP Multiplex Human Cytokine Panel and Obesity Panel, R&D Systems, Minneapolis, USA) and read by the Bioplex 200 array reader (Bio-Rad Laboratories, Hercules, CA, U.S.A.) which uses Luminex xMAP™ Technology (Luminex Corporation, Austin, TX, U.S.A.).

#### Respiratory function and ventilatory responses to CO2

Spirometry and plethysmography were measured according to the European Respiratory Society recommendations [24]. CO_2_ chemo-sensitivity was assessed using Read's method [25].

#### Endothelial Dysfunction

Endothelial dysfunction was assessed by reactive hyperhemia with finger plethysmographic methodology (RH-PAT, i.e Reactive Hyperhemia Peripheral Arterial Tonometry) using Endo-PAT device (Itamar Medical Ltd, Caesarea, Israel) as previously described [26], [27]. RH-PAT index was calculated as the natural logarithm of the average amplitude of PAT signal after 90 to 120 second deflation divided by average amplitude of the PAT signal during 210 second prior the cuff inflation [28].

### Statistical analysis

Our main objective was to unmask differences in endothelial dysfunction between OHS and eucapnic obese patients matched for age and BMI. Our secondary goal was to assess inflammatory parameters differences between the two groups. The first step was to use univariate conditional logistic regression to compare the two groups. Then, multivariate conditional logistic regression models were used to examine the effects of potential confounders other than those controlled for by matching, including parameters unbalanced between groups. For all the tests, a significant level of p<0.05 was used. SAS 9.1.3 package (SAS Institute, Cary, NC, USA) software was used for statistical analysis. Results are expressed as mean±SD.

## Results

### Patients characteristics

Fourteen obesity hypoventilation syndrome patients (OHS) were compared to 39 “eucapnic obese” matched for age and BMI. By definition, PaCO2 was more elevated in OHS than in obese group (6.45±0.39 vs 5.31±0.44 kPa). PaO2 tend to be lower (9.78±1.73 vs 10.71±1.44 kPa). Anthropometrics, blood pressure and lung function characteristics are reported in Table 1. Sex ratio was not different between the two groups. OHS had significantly impaired lung volumes. Compared to eucapnic obese (Table 2), OHS had comparable severity in obstructive sleep apnea syndrome as expressed by apnea+hypopnea index (AHI = 40±28 for UO vs. 57±54 for OHS). Three OHS patients had AHI≤5/h compared to two patients in the eucapnic obese group. OHS spent more time during sleep with oxygen saturation (SpO_2_) less than 90% and had lower nadir nocturnal SpO_2_. As shown in table 3, OHS had higher glycated hemoglobin and tended to be more frequently treated for hypertension. HOMA index reflecting insulin resistance was three time higher in OHS than in eucapnic obese.

**Table 1:** Anthropometric characteristics and respiratory function.

	OHS (14)	Eucapnic Obese (39)	Odds Ratio (95% CI)
Sex F/M	9/5	26/13	0.83 (0.24–2.81)
Age (years)	57±10	56±10	0.99 (0.90–1.09)
BMI (kg/m^2^)	41.0±5.2	40.9±5.1	1.02 (0.85–1.23)
Waist/Hip ratio	0.98±0.06	0.94±0.1	1.52 (0.32–7.15)
Clinical SBP (mmHg)	133±23	132±12	1.00 (0.96–1.05)
Clinical DBP (mmHg)	75±9	80±10	0.96 (0.90–1.02)
FVC (% predicted value)	72±24	92±17 **	0.93 (0.89–0.98)
TLC (% predicted value)	90±17	99±12 §	0.95 (0.90–1.00)
FEV1/FVC (%)	84±8	79±8	0.11 (0.97–1.28)
CO_2_ sensitivity (l/min/mmHg)	1.4±0.9	2.4±1.5 §	0.51 (0.25–1.06)

OHS: Obesity hypoventilation syndrome; BMI: Body Mass index; SBP: Systolic blood pressure; DBP: Diastolic blood pressure; FVC: forced vital capacity, expressed as percentage of predicted value; TLC: total lung capacity as percentage of predicted value; FEV1/FVC: forced expiratory volume in 1 second on forced vital capacity ratio; CO_2_ sensitivity: Central CO_2_ chemo-sensitivity was assessed using Read's method [25]. Results are expressed as mean±SD.

§ : p<0.1; *: p<0.05 **: p<0.01 using univariate conditional logistic regression.

**Table 2:** Sleep structure and sleep associated disorders breathing.

	OHS (14)	Eucapnic Obese (39)	Odds Ratio (95% CI)
Total Sleep Time (min)	341±66	338±83	1.00 (0.99–1.01)
Sleep 1–2 (% of total sleep time)	75±9	72±10	1.05 (0.95–1.15)
Sleep 3–4 (% of total sleep time)	5±8	7±8	0.98 (0.87–1.10)
REM Sleep (% of total sleep time)	19±7	21±8	0.96 (0.87–1.06)
AHI (n/h)	57±54	40±28	1.01 (0.99–1.03)
Respiratory-related μ-arousals(n/h)	50±36	36±20	1.02 (0.99–1.04)
Mean nocturnal SpO2 (%)	89±5	91±4	0.90 (0.78–1.03)
Nadir nocturnal SpO2 (%)	65±15	76±10 *	0.94 (0.89–0.99)
Sleep time spent with SpO2<90% (%)	44±35	19±21 *	1.04 (1.01–1.08)

REM: Rapid eye movement sleep; AHI: Apnea-hypopnea index; SpO_2_: oxygen saturation Results are expressed as mean±SD.

§ : p<0.1; *: p<0.05 using univariate conditional logistic regression.

**Table 3:** Cardiovascular, metabolic status and history.

	OHS (14)	Eucapnic Obese (39)	Odds Ratio (95% CI)
Treated hypertension , *%*	86	54 §	4.74 (0.85–26.53)
Diabetes, *%* (Treated for diabetes, *%*)	54 (43)	29 (21)	3.00 (0.80–11.34)
Statins, *%*	43	23	1.89 (0.50–7.09)
Fast blood insulin level, *μu.mL^−1^*	22.7±21.1	11.2±7.9 §	1.06 (0.99–1.12)
Fast blood glucose level *mmol/l*	7.5±3.8	6.5±2.7	1.15 (0.90–1.48)
HOMA – IR (G*I/22.5)	9.8±13.0	3.2±2.4 §	1.14 (0.98–1.33)
HbA1c, %	7.3±4.3	6.1±1.7 *	8.76 (1.02–75.00)
Triglycerids, *g/l*	1.69±0.8	1.4±0.7	1.75 (0.70–4.40)
HDL cholesterol, *g/l*	0.41±0.13	0.40±0.1	1.51 (0.40–5.72)
LDL cholesterol, *g/l*	1.0±0.4	1.3±0.5 §	0.23 (0.05–1.09)
Total Cholesterol, *g/l*	1.8±0.5	2.0±0.6	0.34 (0.08–1.39)

HOMA-IR was calculated with the formula: Fast blood glucose level*Fast blood insulin level/22.5; HbA1c: Glycated haemoglobin; HDL: High density lipoprotein; LDL: Low density lipoprotein. Results are expressed as mean±SD. ±SD.

§ : p<0.1; *: p<0.05 using univariate conditional logistic regression.

### Inflammatory and anti-inflammatory status

_HS_-CRP was significantly elevated in OHS compared to eucapnic obese (mean±SD: 11.1±10.9 vs. 5.7±5.5 mg.l^−1^, p<0.05). Serum level of RANTES, a pro-atherosclerotic chemokine, was also significantly increased in OHS (55.9±55.3 vs 23.3±15.8 ng/ml, p = 0.04) (table 4). Others pro-inflammatory cytokines, chemokines except MCP1, were not different between the two groups. Adiponectin, an anti-inflammatory adipokine was significantly lowered in OHS compared to UO (7606±2977 vs 13660±7854, ng/ml p = 0.025). (table 4).

**Table 4:** Serum levels of 9 cytokines in Obesity Hypoventilation Syndromes (OHS) compared to “uncomplicated obese” (UO) patients.

	OHS (14)	Eucapnic Obese (39)	Odds Ratio (95% CI)
IL1-Ra, ng/ml	1.6±1.4	1.7±1.9	1.00 (0.99–1.01)
MCP1, pg/ml	172±72	216±76*	0.98 (0.97–0.99)
IL8, pg/ml	6.8±3.4	18 .8±45.3 §	0.88 (0.75–1.02)
TNFa, pg/ml	2.8±2.2	2.7±1.2	1.04 (0.68–1.58)
LEPTIN, ng/ml	92.5±59.2	110.4±82.5	0.99 (0.98–1.01)
IL6, pg/ml-	1.9±1.1	1.7±1.0	1.78 (0.80–3.93)
RESISTIN, ng/ml	6.8±2.8	7.1±4.7	0.96 (0.82–1.13)
RANTES, , ng/ml	55.9±55.3	23.3±15.8 *	1.03 (1.02–1.06)
ADIPONECTIN, ng/ml	7606±2977	13660±7854*	0.15 (0.03–0.70)

§ : p<0.1; *: p<0.05, using univariate conditional logistic regression.

### Endothelial dysfunction

Endothelial function was significantly more impaired in OHS patients than in UO patients (RH-PAT index: 0.22±0.06 versus 0.51±0.11 respectively, odds ratio = 0.02 [0.01–0.48) p = 0.03].

**Figure 1:**
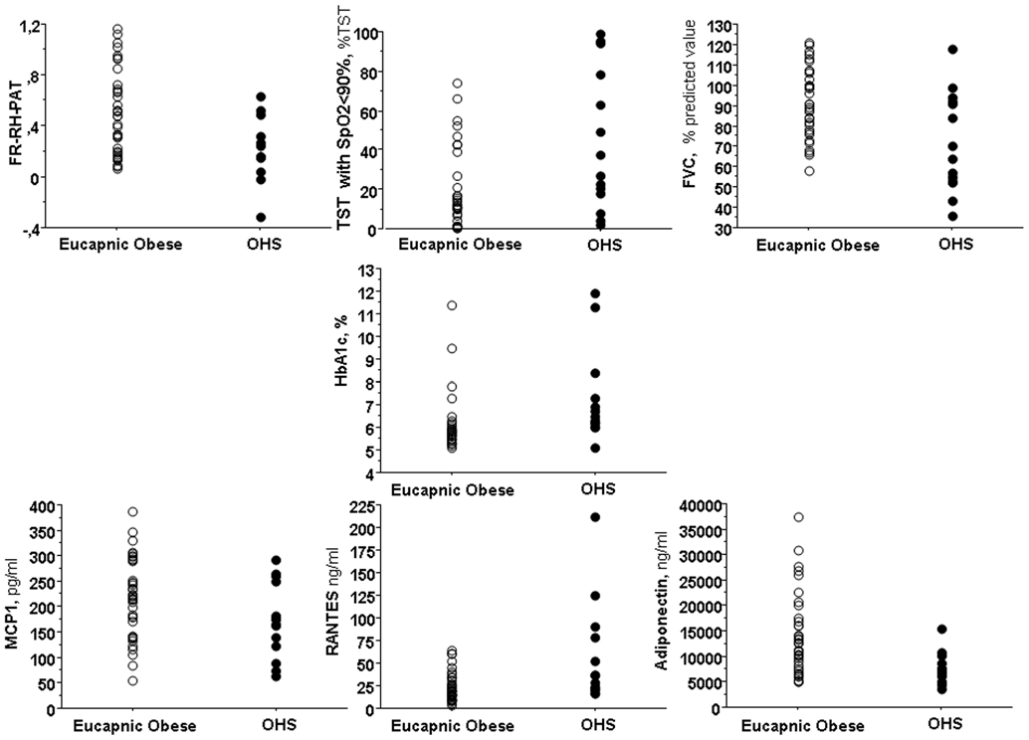
Comparison between Obesity Hypoventilation Syndrome (OHS) compared to eucapnic obese patients in RH-PAT, Sleep time spent with SpO2<90%, FVC (% predicted value), glycated haemoglobin, serum levels of RANTES, Adiponectin and MCP1. TST: total sleep time.

Figure 1 depicts parameters which differed between OHS and eucapnic obsese patients in univariate analysis. In a multivariate analysis, after adjustment for potential confounders (i.e: FVC, Sleep time spent with SpO2<90%, HbA1c), neither RANTES, nor adiponectin and RH-PAT were independently associated with the risk of having OHS.

## Discussion

In this prospective controlled study, we compared for the first time inflammatory status and endothelial function in obesity hypoventilation syndrome and eucapnic obesity. We observed that the proatherogenic chemokine RANTES (CCL5) increased significantly whereas insulin sensitizing and antiatherogenic adipokine adiponectin was significantly reduced in OHS patients. Consistently, endothelial function was significantly more impaired in obesity hypoventilation syndrome than in eucapnic obesity.

Not only obesity but many other systemic diseases like diabetes, COPD, cardiovascular diseases or sleep apnea are associated with an underlying pro-inflammatory state [8], [29], [30]. Although sharing what is generally called ‘low-grade’ or ‘chronic’ inflammation, all these diseases present different time course evolution and prognosis. Moreover, obesity per se, is not a homogeneous condition. It would be useful to distinguish subclasses of inflammation among obese populations reflecting different risks and allowing tailoring specific anti-inflammatory treatments. The present study is the first to compare the serum profile of an extensive panel of 9 chemokines and adipokines by multiplex assay in OHS and eucapnic obese patients. Of all chemokines tested, RANTES was seen to be significantly elevated. RANTES is a chemokine that has been involved in atherogenesis [31], and that is also related to coronary heart disease risk in middle age subjects [32]. Circulating RANTES level is elevated in symptomatic coronary artery disease [33] and is also acutely increased in unstable angina pectoris during severe ischemic symptoms [34]. RANTES is expressed by activated platelets, lymphocytes and adipocytes [35], [36]. As a result circulating RANTES concentrations are elevated in obese rats [37], during human obesity, impaired glucose tolerance and type 2 diabetes [38]. The design of the present study did not allow identifying the main source of plasmatic RANTES but we can speculate on a predominant visceral fat and/or activated platelets origin for RANTES. Indeed, Hosegai et al. [15] have shown that local adipose tissue hypoxia in mice dysregulates adipokines production. In the present study, OHS patients exhibited more severe daytime hypoxaemia and nocturnal oxygen desaturations compared to matched eucapnic obese. Thus, mild hypoxaemia during daytime and severe desaturation during sleep, the later representing an additional hypoxic insult, might favor RANTES production by adipose tissue. On the other hand, thromboembolism is 4 fold increased in mortality in OHS compared to eucapnic obesity [6], suggesting a pro-coagulant state in OHS potentially linked to platelet activation. Platelets are the main source of RANTES and the interplay between RANTES and platelets allow monocytes arrest, a determinant pathway in atherosclerosis initiation [36]. Further studies directly addressing local fat inflammation and platelets activation in the specific population of OHS are required to elucidate the respective contribution of these two pathways in RANTES up-regulation.

Whereas proatherogenic RANTES increased significantly, insulin sensitizing and antiatherogenic adipokine adiponectin was significantly reduced in our OHS patients. Obesity-related cardiovascular diseases are associated with decreased plasma levels of adiponectin [39], [40]. Hypoadiponectinemia correlates significantly and independently with coronary artery disease [41] and more generally plasma adiponectin levels are an inverse predictor of cardiovascular outcome [39]. In our study, as for RANTES, the severity of sleep apnea and REM sleep related desaturations together with moderate daytime hypoxaemia, may play a major role as it has been demonstrated that local hypoxia at the abdominal fat level reduced adiponectin release by the adipocytes [15]. In the present study, we evidenced for the first time that hypoadiponectinemia is further reduced in OHS compared to non OHS obese subjects. This result is in accordance with the OHS observational cohorts data, demonstrating a higher prevalence of cardiovascular diseases as well as increased mortality of OHS patients [2], [6]. We also found that OHS patients tend to used more antihypertensive agents had higher insulin resistance and were more frequently treated by glucose lowering medications. However, strong evidence base medicine is lacking in this field and epidemiological studies in different subclasses of obesity are desirable to more clearly delineate the respective metabolic and cardiovascular risk of different subgroups of obese subjects with and without daytime hypoventilation [42].

As we have demonstrated a specific pattern of inflammation and a decrease in adiponectin levels, we also observed an aggravated endothelial dysfunction in OHS patients [43]. This is supporting a particular cardiovascular risk associated with OHS as endothelial dysfunction is an early key event of atherosclerosis and a strong predictor of incident cardiovascular events [44]–[46]. Arkin et al. [42] have recently suggested that endothelial dysfunction aggravates with increased degree of obesity, being more severe in super obese (>50 kg/m^2^) than in morbidly obese (>40 kg/m^2^). They assumed that more visceral fat in super obese patients can be an explanation for their results. As the expected prevalence of OHS is more than 50% in super obese patients versus only 30% in morbidly obese [6], we suggest that OHS-induced inflammation may be a complementary explanation. This is leading to propose a systematic measurement of blood gazes both in clinical practice and in research protocols in obese. In our study, even supra normal values of PaCO_2_ were significantly linked with RANTES elevation and endothelial dysfunction.

Conditional multivariate analysis did not allow to demonstrate that endothelial dysfunction (i.e: RH-PAT) as well as inflammatory status (i.e: high levels of RANTES/low levels of ADIPONECTIN- MCP1) were independently associated with OHS. Actually, hypoxia both sustained (morbid obesity) and/or intermittent (OSAS) is acknowledged as triggering inflammatory pathways mediated by the transcription factor nuclear factor kappa B (NF-kB) and hypoxia-inducible factor 1 (HIF-1). Particularly, NF-kB is a key player in inflammatory and innate immune responses. Circulatory proinflammatory cytokines are then increased and at the end these inflammatory processes directly induce endothelial dysfunction[47], [48]. Moreover, there is a close relationship between insulin resistance, known to be associated with low serum adiponectin level [49] and endothelial dysfunction[50]. Thus, it appears that inflammatory cytokines, nocturnal desaturation, glycated haemoglobin and endothelial dysfunction form a vicious cycle where each results in worsening of the other.

### Limitations of the study

Several limitations of this study need to be pointed out. Firstly, the sample size of the study (n = 53) is relatively limited. However, in quality assessment before including the studies in the metaanalysis of predicting factors to having OHS, Kaw et al. keep prospective studies with case-control design including at least 10 patients with hypercapnia [1]. Our study corresponds to these criteria. Secondly, owing to the sample size, boundaries for classes of matching criteria were relatively wide. However, the sub-classes of BMI used in the study are corresponding to the classical definitions for moderate to morbid obesity [51].

### Clinical implications, research and therapeutic perspectives

Our study demonstrated that obesity hypoventilation syndrome is a specific cluster in obesity associated with specific inflammation and aggravated endothelial dysfunction. Whether RANTES elevation will allow delineating suitable specific therapeutic targets in this particular subgroup of obese needs to be addressed in future studies [37], [52]. However, this already have clinical implications as individuals with elevated RANTES levels have higher risk to develop diabetes mellitus despite intensive lifestyle intervention than individuals with lower RANTES levels [53]. Moreover, non invasive ventilation the current first line therapy of OHS should now be evaluated, in randomized controlled trials, not only regarding its effects on PaCO_2_, sleep and quality of life but also for its cardiovascular and metabolic impact.
